# Variations in vascular mortality trends, 2001–2010, among 1.3 million women with different lifestyle risk factors for the disease

**DOI:** 10.1177/2047487314563710

**Published:** 2015-12

**Authors:** Benjamin J Cairns, Angela Balkwill, Dexter Canoy, Jane Green, Gillian K Reeves, Valerie Beral, for the Million Women Study Collaborators

**Affiliations:** Cancer Epidemiology Unit, University of Oxford, Oxford, UK

**Keywords:** Coronary heart disease, cerebrovascular disease, mortality, secular trends, smoking, obesity

## Abstract

**Aims:**

Vascular disease mortality has declined rapidly in most Western countries, against a background of improved treatments and falling prevalence of smoking, but rising obesity. We examined whether this decline differed by lifestyle risk factors for vascular disease.

**Methods and Results:**

During 2001–2010, there were 9241 vascular disease deaths in a prospective study of 1.3 million women in middle age, about one-quarter of all UK women in the eligible age range (50–64 years in 1996–2001). We estimated percentage declines in mortality from coronary heart disease, cerebrovascular disease and other vascular diseases, overall and by age, smoking, alcohol consumption, adiposity, physical activity, socioeconomic status and age at leaving school. Over 10 years, coronary heart disease mortality fell by half (52%), cerebrovascular disease mortality by two-fifths (42%) and other vascular disease mortality by one-fifth (22%). Lean women experienced greater declines in coronary heart disease mortality than overweight or obese women (70%, 48% and 26%, respectively; *P* < 0.001 for heterogeneity) and women in the highest and middle thirds of socioeconomic status experienced greater declines in other (non-coronary, non-cerebrovascular) vascular disease mortality than women in the lowest third (41% and 42% and –9%, respectively; *P* = 0.001). After accounting for multiple testing, there were no other significant differences in vascular mortality trends by any lifestyle risk factor, including by smoking status.

**Conclusion:**

Vascular disease mortality trends varied in this cohort by adiposity and socioeconomic status, but not by smoking status or other lifestyle risk factors. Prevention and treatment of vascular disease appear not to have been equally effective in all subgroups of UK women.

## Introduction

Vascular disease mortality has fallen greatly since the 1950s in both men and women in the UK, the USA and many other countries.^1^ The causes of this decline include improved treatments and changes over time in important risk factors such as smoking, blood pressure and cholesterol.^2–5^ However, most of what is known about these trends is derived from population-level data. Although some prospective studies have investigated socioeconomic differences in declining vascular mortality,^6–12^ few have examined whether the decline has varied by specific, individual-level lifestyle characteristics.^8,13–18^

Two trends in lifestyle risk factors are of particular relevance to trends in vascular mortality. First, smoking cessation has been an important driver of the decline in vascular mortality,^19,20^ but never-smokers cannot stop smoking. The causes of declining vascular mortality that act on never-smokers will therefore differ from those acting on smokers, but the ways in which they differ, and their net effects in each group, are presently unknown. Second, there is evidence that rising obesity might have slowed the decline in vascular mortality in some populations,^3,21,22^ but it is unknown whether within these populations lean and obese adults have experienced similar declines in vascular mortality. The aim of this study was to systematically test whether the most recent declines in vascular mortality differed by individual lifestyle characteristics that are associated with vascular disease risk, using individual-participant data from a large prospective cohort of UK women.

## Methods

Between 1996 and 2001, 1.3 million women were recruited through the National Health Service breast screening programme in England and Scotland, about a quarter of UK women in the target age range during the recruitment period. Each woman responded to a recruitment questionnaire, providing information on a range of health and lifestyle factors; 96% were aged 50–64 years at recruitment. Study questionnaires and further details of the data and access policies can be viewed on the website (www.millionwomenstudy.org). All participants gave written informed consent to take part; ethical approval was provided by the Oxford and Anglia Multi-Centre Research and Ethics Committee.

All participants are routinely followed up by linkage to UK Office of National Statistics death and emigration records, providing investigators with dates of deaths, along with underlying and contributing causes coded according to the International Classification of Diseases, 10th Revision (ICD-10). We examined deaths with a vascular disease as the underlying cause (codes I00–I99). Analyses were also performed for deaths attributed to all coronary heart disease (I20–I25) and, separately, myocardial infarction (I21–I22) and other coronary heart disease (I20, I23–I25), cerebrovascular disease (I60–I69) and all other vascular diseases (I00–I19, I26–I59, I70–I99).

### Statistical analysis

Hazard ratios of death attributed to vascular disease were estimated by Cox proportional hazards regression, with attained age as the underlying time variable, which allowed very fine adjustment for age in all analyses. Follow-up began for each woman on 1 January 2001, or 4 years after her date of recruitment, whichever came last. Limiting the follow-up period to begin no sooner than 1 January 2001 ensured that cause of death coding was relatively consistent in our analyses. On this date, underlying cause of death coding was changed in England and Wales from ICD-9 to ICD-10, which could have biased our estimates by producing artefactual jumps in mortality from certain diseases: compared to ICD-10, ICD-9 coding rules tended to ascribe more deaths to myocardial infarction and pneumonia and fewer to stroke.^23,24^ We additionally excluded the first 4 years of follow-up after the date of recruitment in case women who were already ill, and at greater risk of death soon after recruitment, had experienced changes to lifestyle risk factors as a result of their illness (reverse causation). The last date of follow-up was, for each woman, her date of death, emigration or other loss to follow-up, or 31 December 2010, whichever came first. Follow-up for each woman was censored at her 70th birthday, so that results represent trends in vascular mortality for women in late middle age.

To calculate absolute vascular mortality rates per 100,000 women, hazard ratios were first estimated within 2-year time periods (2001–2002, 2003--2004, 2005--2006, 2007--2008, 2009–2010). Variances of log risks and 95% confidence intervals (CI) were calculated using the method of floating absolute risks.^25^ A technique similar to that described by the Prospective Studies Collaboration^26^ was used to put these hazard ratios on a representative absolute scale: the hazard ratios and 95% CI were multiplied by the overall uniformly age-standardized vascular mortality rate for the entire cohort, divided by a weighted average of the time period-specific hazard ratios (weights were given by the total person-time at risk during each time period). Whether or not these hazard ratios deviated from log-linearity was assessed using a likelihood ratio test.

Trends over time in mortality rates were estimated by Cox regression, treating calendar year as a continuous variable, and were expressed as percentage declines per 1 or per 10 years. Negative percentage declines indicate increases in vascular mortality over time. Percentage declines were estimated at each level of individual characteristics reported at baseline: attained age (<60, 60–64, 65–69 years); smoking status (never, former, current smoker); alcohol consumption (<1, 1–6, 7+ drinks per week, where a drink contains approximately 10 g of ethanol); body mass index (BMI; <25, 25–29.9, 30+ kg/m^2^); frequency of strenuous exercise (rarely/never, at most once, more than once per week); socioeconomic status (thirds of the Townsend deprivation index,^27^ a standardized measure based on statistics on unemployment, car ownership, home ownership and household overcrowding, derived from 1991 UK census data for the local area of each woman’s home address at recruitment); age at leaving school (<16, 16, 17+ years). Heterogeneity of trends in mortality rates was assessed using a chi-squared contrast test. To compare results from the Million Women Study cohort with population-wide trends, we extracted death and population data from the WHO Mortality Database.^28^ We used Poisson regression to estimate trends from 2001–2010 in vascular disease mortality, for UK women aged 50–69 years.

All analyses were stratified by region and year of recruitment to account for differences in vascular disease mortality due to recruitment-related cohort effects. Risk factor status was not updated over time, due to incomplete response to resurveys, and in the main analyses no adjustments were made for other covariates. Our estimates therefore quantified the overall vascular mortality trends within subgroups of each risk factor, rather than the residual trends that remained unexplained after accounting for other factors. For example, estimated trends would include any effect of smoking cessation on vascular mortality rates. We examined potential for confounding at baseline by tabulating baseline characteristics for women at risk in different periods of calendar time and in a sensitivity analysis, which was adjusted for all covariates. Women who reported prior vascular disease at recruitment (ever had or now being treated for heart disease, stroke and blood clots or clotting problems) were included in the main analysis, but excluded from a sensitivity analysis to assess reverse causation by pre-existing illness.

All statistical tests were two-sided. Multiple testing was accounted for using the Holm-Bonferroni method for the 21 tests in the main analysis: for any test to be considered statistically significant, at least one *P*-value must be <0.05/21≈0.0024.^29^ All analyses were conducted using Stata, version 12.1 (StataCorp, Texas).

### Role of the funding source

The study sponsors had no involvement in the conduct of the study, the writing of this report or the decision to submit for publication.

## Results

From 1 January 2001 to 31 December 2010, there were 9241 deaths with a vascular disease as the underlying cause among 1,312,656 women, with a total follow-up time of 8.7 million years. The women were aged 56.4 years on average at baseline (Table 1). As expected, those who died of vascular causes were, on average, slightly older at baseline than those who did not, had a higher BMI, drank less alcohol, were less likely to engage in strenuous physical activity and were more likely to be current smokers, to have lower socioeconomic status and to have left school at an earlier age. Characteristics of the women varied little with the time period in which they were at risk, after adjustment for region, year and age at recruitment (see Table 1 in Supplementary Material), indicating little potential for residual confounding in analyses stratified by these factors.

**Table 1.** Characteristics of participants at baseline.

		Vascular disease mortality during follow-up (ICD-10 codes)			
	All eligible participants	Did not die of a vascular disease	Coronary heart disease deaths (I20–I25)	Cerebrovascular disease deaths (I60–I69)	All other vascular disease deaths (I00–I19, I26–I59, I70–I99)
	*n* = 1,312,656	*n* = 1,303,415	*n* = 4269	*n* = 2317	*n* = 2655
Characteristic at baseline					
Age, mean (SD), years	56.4 (4.5)	56.4 (4.5)	57.1 (4.0)	56.8 (4.0)	56.8 (4.0)
Body mass index, mean (SD), kg/m^2^	26.2 (4.7)	26.2 (4.7)	28.3 (6.2)	26.5 (5.5)	28.3 (6.5)
Alcohol, mean (SD), drinks/week	2.0 (0.7)	2.0 (0.7)	1.7 (0.7)	1.9 (0.7)	1.8 (0.7)
Smoking, % current	20.5	20.3	49.1	44.2	36.6
Strenuous exercise, % inactive^a^	48.4	48.3	67.3	62.5	64.9
Socioeconomic status, % lower third	33.2	33.1	53.2	45.1	48.4
Age at leaving school, % <16 years	54.7	54.6	70.3	64.6	65.7

*n*: number of women.

a Inactive women reported ‘rarely/never’ doing strenuous exercise.

**Figure 1.**
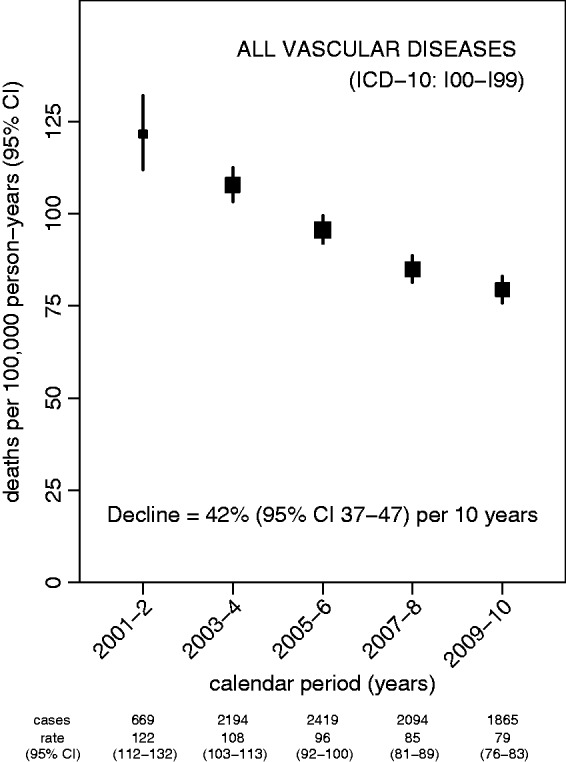
Mortality attributed to vascular diseases, 2001–2010, and the decline in vascular mortality during the same period.

**Figure 2.**
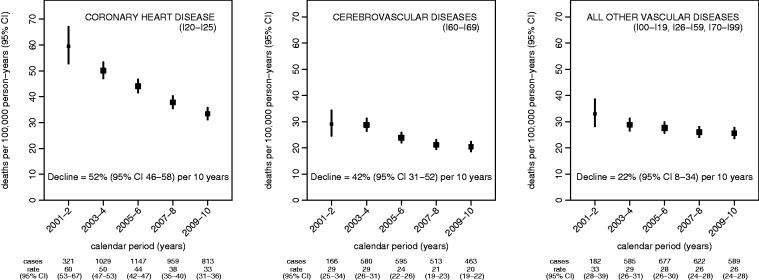
Mortality attributed to specific categories of vascular diseases (ICD-10 codes), 2001–2010, and corresponding declines in mortality during the same period.

Vascular disease mortality showed clear evidence of a sustained decline between 2001 and 2010 (Figure 1). The relative decline in vascular mortality during this 10-year period was 42% overall (95% CI 37–47%; *P* < 0.001), but varied by specific cause of death (Figure 2). Coronary heart disease mortality declined most strongly, by 52% over 10 years (46–58%; *P* < 0.001), followed by cerebrovascular mortality (42% over 10 years, 31–52%; *P* < 0.001) and mortality from all other vascular diseases combined (22% over 10 years, 8–34%; *P* = 0.004). There was no evidence that the relative decline in mortality varied over this period for all vascular diseases combined (*P* = 0.6 for test of nonlinearity), or separately for coronary heart disease (*P* = 0.9), cerebrovascular disease (*P* = 0.3) or other vascular diseases (*P* = 0.7). Estimated declines in vascular mortality between 2001 and 2010 for all UK women 50–69 years, derived from the WHO Mortality Database, were 56% for coronary heart disease, 49% for cerebrovascular disease and 28% for other vascular diseases; each was within the confidence limits of the estimates from the Million Women Study.

**Figure 3.**
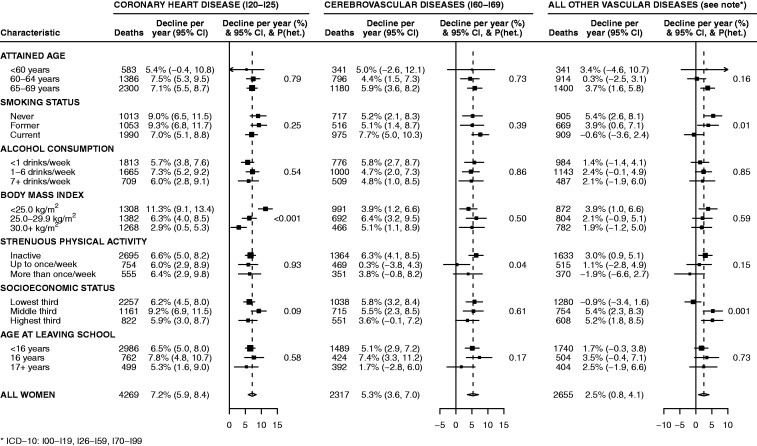
Annual declines in mortality attributed to specific categories of vascular diseases (ICD-10 codes), 2001–2010, by various characteristics reported at baseline.

Figure 3 gives estimated annual relative declines in coronary heart disease mortality, cerebrovascular mortality and other vascular mortality by baseline characteristics of the women. For coronary heart disease (*n* = 4269 deaths), mortality fell significantly more among lean (BMI < 25 kg/m^2^) than overweight (BMI 25–29.9 kg/m^2^) or obese (BMI ≥ 30 kg/m^2^) women, equivalent to declines per 10 years of 70%, 48% and 26%, respectively (*P* < 0.001 for heterogeneity). Declines in coronary heart disease mortality did not otherwise vary by any characteristics, including smoking status (all *P* > 0.05). The decline in cerebrovascular disease mortality (*n* = 2317 deaths) did not vary significantly by any characteristic, after adjustment for multiple testing (all *P* ≥ 0.04).

There were 2655 deaths from other vascular diseases, which included a range of conditions, the most common being venous thromboembolism, heart failure and other heart disease, and aortic aneurysm (31%, 11%, and 13% of these deaths, respectively). Overall, after adjustment for multiple testing there were significantly greater declines in other vascular disease mortality among women in the highest and middle thirds than the lowest third of socioeconomic status (equivalent declines over 10 years of 41%, 42% and –9%, respectively; *P* = 0.001). There was weak (non-significant) evidence that other vascular disease mortality declined more among never and past smokers than among current smokers (equivalent declines over 10 years of 43%, 33% and –6%, respectively; *P* = 0.01 for heterogeneity, non-significant after adjustment for multiple testing).

The relative decline in mortality due to all vascular diseases combined varied, as expected, in the same ways as the component causes (see Figure 1 in Supplementary Material) and most strongly by BMI (*P* = 0.002 for heterogeneity). Declines in mortality from myocardial infarction and from other coronary heart disease each varied according to BMI (*P* < 0.001 and *P* = 0.01, respectively)

Results of sensitivity analyses were similar to those of the main analysis. None of the findings described above was materially changed by additional adjustment for other baseline characteristics (all *P*-values were similar in magnitude and statistically significant as per the minimally adjusted main analysis). After exclusion of women who reported prior vascular disease, there were smaller relative declines in vascular mortality in most subgroups, but similar patterns by lifestyle risk factors (see Figure 2 in Supplementary Material).

## Discussion

The fall in vascular mortality is one of the most striking health achievements of recent decades, driven both by beneficial trends in risk factors such as smoking and by improved treatments.^2–5^ However, there are signs that this trend may be slowing in some populations, for example due to adverse trends in obesity and diabetes.^21,22^ Despite the knowledge that there are substantial variations in vascular disease risk and risk factors within populations, there has been limited research into whether trends in vascular mortality also vary. Large cohort studies can be used to investigate these variations and to identify which subgroups might stand to benefit most from interventions to prevent or treat vascular disease.

In this cohort, the decline in vascular mortality was similar in many, but not all subgroups of UK women. During the 10-year period 2001–2010, overall vascular disease mortality declined by almost half. Coronary heart disease mortality declined by 52%, but (after accounting for multiple testing) by significantly more among lean than obese women. Cerebrovascular disease mortality declined by 42%. Mortality from other vascular diseases declined by only 22%, but by significantly more among the higher socioeconomic groups than the lowest. Previous studies have shown that changes in treatments and in prevalence of risk factors can each explain up to half of the observed decline in vascular mortality at the population level, indicating that there are multiple causes of these trends.^2–5^ Our results therefore suggest that the cumulative effects of these causes have varied between different groups of women.

Our strongest finding was that there were faster declines in coronary heart disease mortality among lean women (BMI < 25 kg/m^2^) than among overweight (BMI 25–29.9 kg/m^2^) or obese women (BMI ≥ 30 kg/m^2^). The 10-year relative decline in coronary heart disease mortality was 70% in lean women, 48% in overweight women and only 26% in obese women. The reasons for these differences are unknown and data were not available in this cohort to test specific hypotheses. However, in the UK obesity has a greater prevalence in women with a lower socioeconomic position,^30,31^ suggesting that there may also be differences in dietary habits, propensity to seek treatment for symptoms and other behaviours, by obesity status. Overweight and obese women might therefore have experienced smaller benefits from improved treatments or population-level reductions in risk factors, such as high cholesterol or blood pressure. In this cohort, the approximately 1 kg/m^2^ per decade increase in mean BMI in UK women^32^ would, by itself, have had relatively small effects on vascular mortality during follow-up. However, longer-term trends in obesity might be related to access to or utilisation of specific medical and surgical treatments, as well as other clinical risk factors, such as diabetes (the prevalence of which has continued to increase in UK women).^33^

Because prior disease can cause both weight loss and death, reverse causation is an important potential source of bias in estimates of associations between obesity and mortality^26^ and could, in principle, generate effects similar to those we observed. However, the first 4 years of follow-up were excluded from all analyses, and a sensitivity analysis in which we excluded women who reported prior vascular disease still showed substantially greater declines in coronary heart disease mortality among lean women. We also observed no significant differences by BMI in the declines in cerebrovascular and other vascular mortality. This suggests that the association with obesity is specific to trends in coronary heart disease mortality, despite these other causes of death sharing important risk factors and some medical treatments with coronary heart disease. These results are not directly comparable to the limited evidence from other studies, which investigated other populations and earlier time periods. In the USA, the excess risk of overall vascular mortality may have declined faster between the 1970s and early 2000s among adults with higher BMI,^16,17^ while in two studies of Israeli men, coronary heart disease mortality trends between the 1960s and 1990s did not differ by BMI.^8,15^

We expected that the decline in vascular mortality might differ by smoking status, because women who were current smokers at baseline could have stopped smoking and past smokers may still be gaining the benefits of having stopped. However, we found little evidence of such differences for overall coronary heart disease mortality and for cerebrovascular mortality and, at most, weak evidence of a greater decline among never than current smokers for mortality from other vascular diseases. Never-smokers cannot gain any direct benefits of stopping smoking and so the similarity of the declines in vascular mortality by smoking status suggests that never-smokers may have benefitted more than past or among current smokers from factors other than smoking cessation. Reverse causation could again play a role, although we found no evidence of it in our sensitivity analysis excluding prior disease. As with obesity, smoking and smoking cessation are strongly socially patterned^34^ and other health behaviours associated with smoking may influence how quickly smokers benefit from changes in treatments and risk factors. A comparison of three large US cohorts found that over the last 50 years the relative risks of death due to coronary heart disease and cerebrovascular disease have increased for current compared to never-smokers, with relative risks for women converging to those for men as cigarette technology and women’s smoking behaviours have changed.^18^ Because the health effects of smoking are the consequence of exposure to tobacco smoke over a long period of time, long-term changes in smoking behaviour or cigarette technology are less likely to explain the trends over the 10-year duration of our study.

We found strong evidence that the decline in mortality was greater in women in the higher socioeconomic groups, but only for other (non-coronary, non-cerebrovascular) vascular diseases. This could arise if inequalities in access to treatments for these other vascular diseases have increased, but is difficult to explain by socioeconomic inequalities in risk factor trends. Trends in risk factors such as smoking may have been less favourable for adults in more deprived socioeconomic groups,^34^ but this would be expected to also affect declines in mortality due to coronary heart disease and cerebrovascular disease. Allowing for statistical uncertainty, our findings are not inconsistent with evidence that relative inequalities in coronary heart disease and cerebrovascular disease mortality may have widened a little recently among UK women,^35^ but not with the stronger trends towards greater inequalities reported for some other countries.^6,7,9,11,12,36,37^ We found no evidence that age at leaving school was an important modifier of the decline in vascular mortality in these UK women. Previous cohort studies of vascular mortality trends by educational attainment in comparable Western countries have often but not always found greater declines in vascular mortality among individuals with a higher level of education.^6,7,9,10,36^

For other lifestyle risk factors, we found no evidence of variations in the declines in vascular mortality (other conventionally significant associations in this study could be due to chance). Evidence from other studies on variations by physical activity level is limited to one study of Israeli men, which reported no difference in declining vascular mortality between the 1960s and 1990s by leisure-time physical activity.^8^ Secular trends in alcohol consumption have been linked to large fluctuations in mortality in Russia, including mortality from coronary heart disease other than myocardial infarction,^38^ but no prospective studies of which we are aware have investigated whether vascular mortality trends might have varied by baseline alcohol consumption.

### Limitations and strengths

Despite its large size and virtually complete follow-up for deaths, this study has several limitations. In general, our findings may provide a useful comparison to other populations, but cannot be directly applied to other countries, to men or to other periods of time. Because women were recruited to the Million Women Study via the UK national breast screening programme, they also tend to have a somewhat higher socioeconomic status than the general population.^39^ We could not investigate women who did not attend screening or those who declined to participate in the Million Women Study, although UK-wide trends in vascular mortality among women of a similar age were within the confidence limits of our estimates. We were unable to investigate the roles of established risk factors such as blood pressure, lipids and diabetes mellitus, or of treatments to manage these factors, which will be important as probable proximate causes of the observed differences in the declines in vascular mortality. Because this is an observational study, it is also impossible to completely rule out effects of pre-existing disease and other sources of confounding. However, to minimize potential biases we stratified our analyses by recruitment-related factors, excluded the first 4 years of follow-up and restricted follow-up to a period in which relatively uniform rules were used to assign an underlying cause of death.

## Conclusion

It is not known whether the recent, substantial decline in vascular mortality can be sustained, and if so, how and among whom. Our study suggests that there may be previously unknown differences in this decline between some groups of UK women. In particular, coronary heart disease mortality may have declined more in lean than in overweight and obese women, and never-smokers may have benefitted more than past or current smokers from drivers of declining vascular mortality other than smoking cessation. A focus of prevention in the groups which have benefitted least should be on weight loss and smoking cessation, respectively, but our findings highlight the need to consider potential differences in other risk factors and access to treatments for vascular disease between major population subgroups. It will be important to discover whether those who appear to have experienced the smallest declines in the past can see greater improvements in the future, through better-targeted prevention and treatment.

## Supplementary Material

Supplementary material
