# Supplementary material for: Variations in vascular mortality trends, 2001–2010, among 1.3 million women with different lifestyle risk factors for the disease
Source: Eur J Prev Cardiol. 2015 Dec;22(12):1626–34. doi: 10.1177/2047487314563710 (PMC4639812; doi:10.1177/2047487314563710)
Supplement: Supplementary material [file Supplementary_Material.pdf]

Variations in vascular mortality trends, 2001-2010, among 1.3 million women with different lifestyle risk factors for the disease

SUPPLEMENTARY MATERIAL

Supplementary Table 1

Supplementary Figure 1

Supplementary Figure 2

Supplementary Acknowledgements

Benjamin J. Cairns,[1,a] Angela Balkwill,[1] Dexter Canoy,[1] Jane Green,[1] Gillian K. Reeves,[1] and Valerie Beral,[1] for the Million Women Study Collaborators

[1] Cancer Epidemiology Unit, Nuffield Department of Population Health, University of Oxford, Richard Doll Building, Roosevelt Drive, Oxford OX3 7LF, United Kingdom.

[a] Corresponding author. Phone: +44 (0)1865 289 600; E-mail: ben.cairns@ceu.ox.ac.uk

Supplementary Table 1. Adjusted characteristics at baseline, duration of follow-up, and vascular deaths among women at risk, by calendar time period

	Time period (calendar years)				
	**2001-2002**	**2003-2004**	**2005-2006**	**2007-2008**	**2009-2010**
	n = 704,499	n = 1,290,223	n = 1,218,243	n = 1,076,463	n = 923,506
**Characteristic at baseline**					
Body mass index, mean (SD), kg/m^2	26.3 (4.6)	26.3 (4.7)	26.3 (4.7)	26.2 (4.7)	26.2 (4.7)
Alcohol, mean (SD), drinks/week	1.9 (0.7)	1.9 (0.7)	1.9 (0.7)	1.9 (0.7)	1.9 (0.7)
Smoking, % current	14.4	14.5	14.3	14.4	14.8
Strenuous exercise, % inactive [a]	48.2	47.7	47.5	47.1	47.9
Socioeconomic status, % lower third	26.8	27.4	27.3	26.9	26.3
Age at leaving school, % <16 years	54.6	55.3	55.3	55.9	57.4
**Follow-up for vascular mortality**					
Person-years, millions	0.6	2.1	2.3	2.0	1.7
Vascular deaths (ICD-10: I00-I99), n	669	2194	2419	2094	1865

Abbreviations: n - number of women at risk

All values except person-years of follow-up and the number of vascular deaths were adjusted for age at baseline, and region and calendar year of recruitment.

[a] Inactive women reported "rarely/never" doing strenuous exercise

Characteristic	ALL VASCULAR DISEASES (I00–I99) Deaths	Decline per year (95% CI)	P(het.)	MYOCARDIAL INFARCTION (I21–I22) Deaths	Decline per year (95% CI)	P(het.)	OTHER CORONARY HEART DISEASE (see note*) Deaths	Decline per year (95% CI)	P(het.)
**ATTAINED AGE**									
<60 years	1265	4.8% (0.8, 8.5)		259	10.4% (2.1, 17.9)		324	1.1% (−7.1, 8.7)	
60−64 years	3096	4.7% (3.2, 6.1)	0.40	607	8.3% (5.1, 11.4)	0.47	779	6.8% (4.0, 9.6)	0.20
65−69 years	4880	5.9% (4.7, 7.0)		1044	10.7% (8.4, 13.0)		1256	4.1% (1.8, 6.3)	
**SMOKING STATUS**									
Never	2635	6.8% (5.2, 8.4)		450	8.2% (4.4, 11.9)		563	9.7% (6.3, 12.9)	
Former	2238	6.7% (5.0, 8.4)	0.33	460	12.1% (8.4, 15.6)	0.32	593	7.0% (3.7, 10.3)	0.01
Current	3874	5.4% (4.1, 6.8)		905	10.9% (8.3, 13.5)		1085	3.5% (0.8, 6.1)	
**ALCOHOL CONSUMPTION**									
<1 drinks/week	3573	4.6% (3.2, 5.9)		820	6.3% (3.4, 9.1)		993	5.3% (2.6, 7.9)	
1−6 drinks/week	3808	5.2% (3.8, 6.5)	0.81	742	12.3% (9.3, 15.1)	0.02	923	3.1% (0.3, 5.9)	0.52
7+ drinks/week	1705	4.5% (2.5, 6.6)		304	9.1% (4.4, 13.6)		405	3.6% (−0.9, 7.8)	
**BODY MASS INDEX**									
<25.0 kg/m^2	3171	7.0% (5.6, 8.5)		599	14.5% (11.3, 17.5)		709	8.6% (5.5, 11.5)	
25.0–29.9 kg/m^2	2878	5.2% (3.6, 6.7)	0.002	659	8.3% (5.1, 11.4)	<0.001	723	4.4% (1.2, 7.4)	0.01
30.0+ kg/m^2	2516	3.0% (1.3, 4.7)		538	4.4% (0.7, 8.0)		730	1.8% (−1.4, 5.0)	
**STRENUOUS PHYSICAL ACTIVITY**									
Inactive	5692	5.5% (4.4, 6.6)		1234	8.4% (6.1, 10.7)		1461	5.1% (2.9, 7.3)	
Up to once/week	1738	3.1% (1.0, 5.1)	0.05	326	7.2% (2.6, 11.6)	0.19	428	5.0% (0.9, 8.9)	0.36
More than once/week	1276	3.4% (1.0, 5.7)		233	13.1% (7.9, 18.1)		322	1.4% (−3.5, 6.1)	
**SOCIOECONOMIC STATUS**									
Lowest third	4575	4.2% (3.0, 5.5)		973	8.4% (5.8, 11.0)		1284	4.5% (2.2, 6.9)	
Middle third	2630	7.1% (5.5, 8.7)	0.02	576	11.8% (8.5, 15.0)	0.28	585	6.7% (3.3, 10.0)	0.41
Highest third	1981	5.1% (3.2, 6.9)		346	9.1% (4.8, 13.3)		476	3.4% (−0.5, 7.2)	
**AGE AT LEAVING SCHOOL**									
<16 years	6215	4.9% (3.8, 5.9)		1341	9.0% (6.8, 11.2)		1645	4.4% (2.3, 6.5)	
16 years	1690	6.4% (4.4, 8.4)	0.15	354	10.9% (6.5, 15.0)	0.73	408	5.2% (1.0, 9.2)	0.78
17+ years	1295	3.4% (1.0, 5.7)		208	8.6% (2.9, 13.9)		291	2.9% (−2.2, 7.8)	
**ALL WOMEN**	9241	5.4% (4.5, 6.2)		1910	9.9% (8.0, 11.7)		2359	4.9% (3.2, 6.6)	

Decline per year (%) & 95% CI, & P(het.):

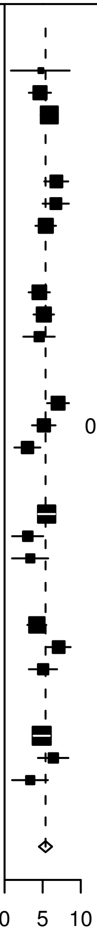
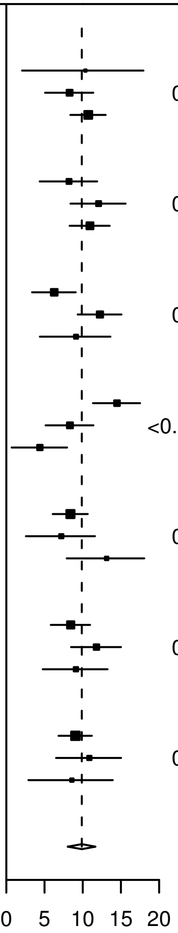
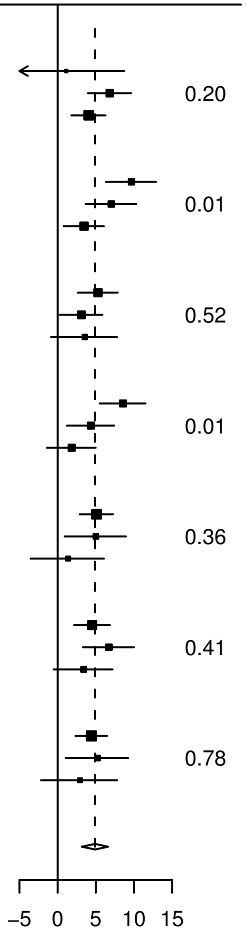

* ICD−10: I20, I23−I25

Supplementary Figure 1. Annual declines in mortality attributed to all vascular diseases, myocardial infarction, and other coronary heart disease, 2001-2010, by various characteristics reported at baseline

Characteristic	CORONARY HEART DISEASE (I20–I25) Deaths	Decline per year (95% CI)	P(het.)	CEREBROVASCULAR DISEASES (I60–I69) Deaths	Decline per year (95% CI)	P(het.)	ALL OTHER VASCULAR DISEASES (see note*) Deaths	Decline per year (95% CI)	P(het.)
**ATTAINED AGE**									
<60 years	433	5.7% (−1.1, 12.0)		290	4.4% (−4.0, 12.2)		249	1.5% (−8.0, 10.2)	
60–64 years	982	5.7% (3.1, 8.2)	0.89	624	3.0% (−0.3, 6.3)	0.55	696	−1.7% (−5.1, 1.6)	0.31
65–69 years	1519	4.9% (2.9, 6.9)		893	5.4% (2.7, 7.9)		958	1.6% (−1.1, 4.1)	
**SMOKING STATUS**									
Never	740	7.0% (4.0, 9.9)		577	2.8% (−0.8, 6.3)		686	2.8% (−0.6, 6.0)	
Former	572	8.1% (4.7, 11.5)	0.33	372	3.6% (−0.8, 7.9)	0.04	419	2.6% (−1.7, 6.6)	0.07
Current	1479	5.3% (3.1, 7.4)		781	8.4% (5.4, 11.3)		677	−2.4% (−5.9, 1.1)	
**ALCOHOL CONSUMPTION**									
<1 drinks/week	1141	3.7% (1.2, 6.1)		565	4.6% (1.0, 8.0)		663	0.0% (−3.5, 3.3)	
1–6 drinks/week	1190	5.1% (2.6, 7.4)	0.68	793	4.0% (1.0, 7.0)	0.97	837	−0.2% (−3.3, 2.8)	1.00
7+ drinks/week	548	5.2% (1.5, 8.8)		428	4.0% (−0.2, 8.1)		372	−0.2% (−4.9, 4.3)	
**BODY MASS INDEX**									
<25.0 kg/m^2	961	10.3% (7.8, 12.8)		814	2.5% (−0.6, 5.5)		636	0.3% (−3.2, 3.7)	
25.0–29.9 kg/m^2	971	3.3% (0.5, 6.0)	<0.001	545	7.0% (3.4, 10.4)	0.16	584	1.5% (−2.2, 5.0)	0.78
30.0+ kg/m^2	782	0.3% (−2.9, 3.4)		321	4.5% (−0.4, 9.1)		535	−0.4% (−4.3, 3.4)	
**STRENUOUS PHYSICAL ACTIVITY**									
Inactive	1767	4.7% (2.6, 6.6)		1021	6.1% (3.5, 8.7)		1091	0.6% (−2.1, 3.2)	
Up to once/week	564	3.8% (0.1, 7.3)	0.80	393	−1.4% (−6.1, 3.1)	0.02	408	−0.1% (−4.5, 4.2)	0.62
More than once/week	422	5.6% (1.5, 9.5)		297	3.2% (−1.9, 8.0)		304	−2.3% (−7.6, 2.8)	
**SOCIOECONOMIC STATUS**									
Lowest third	1465	4.9% (2.6, 7.0)		775	6.0% (3.0, 8.9)		883	−3.8% (−6.9, −0.7)	
Middle third	824	7.2% (4.3, 10.0)	0.15	571	3.5% (−0.1, 7.0)	0.28	548	3.0% (−0.7, 6.5)	0.001
Highest third	626	2.8% (−0.6, 6.2)		451	2.1% (−2.0, 6.1)		461	4.4% (0.6, 8.2)	
**AGE AT LEAVING SCHOOL**									
<16 years	1975	5.1% (3.3, 7.0)		1134	4.3% (1.8, 6.8)		1216	−0.6% (−3.1, 1.9)	
16 years	549	4.4% (0.7, 8.0)	0.71	348	6.9% (2.4, 11.3)	0.12	361	1.7% (−2.9, 6.1)	0.68
17+ years	392	3.3% (−1.1, 7.5)		316	0.0% (−5.1, 4.9)		321	0.2% (−4.8, 5.0)	
**ALL WOMEN**	2934	5.2% (3.7, 6.8)		1807	4.4% (2.4, 6.4)		1903	0.3% (−1.7, 2.3)	

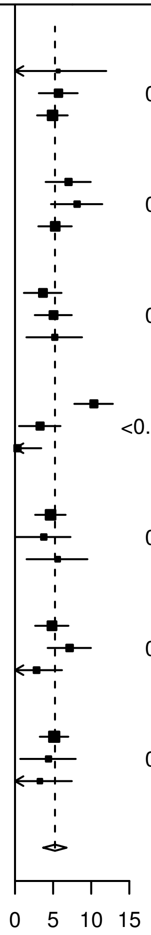

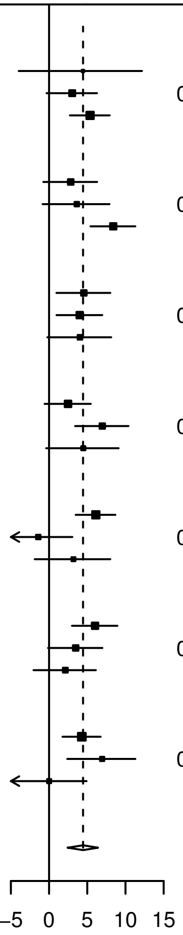

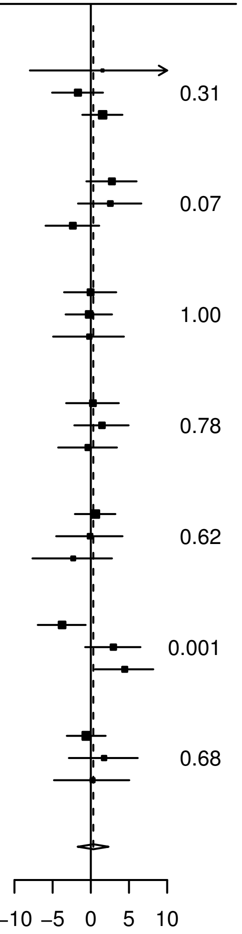

* ICD−10: I00−I19, I26−I59, I70−I99

Supplementary Figure 2. Annual declines in mortality attributed to coronary heart disease, cerebrovascular disease, and other vascular disease (ICD-10 codes), 2001-2010, by various characteristics reported at baseline, excluding women who reported a prior history of vascular disease

Supplementary Acknowledgements

Collaborating NHS Breast Screening Centres. Avon, Aylesbury, Barnsley, Basingstoke, Bedfordshire & Hertfordshire, Cambridge & Huntingdon, Chelmsford & Colchester, Chester, Cornwall, Crewe, Cumbria, Doncaster, Dorset, East Berkshire, East Cheshire, East Devon, East of Scotland, East Suffolk, East Sussex, Gateshead, Gloucestershire, Great Yarmouth, Hereford & Worcester, Kent (Canterbury, Rochester, Maidstone), Kings Lynn, Leicestershire, Liverpool, Manchester, Milton Keynes, Newcastle, North Birmingham, North East Scotland, North Lancashire, North Middlesex, North Nottingham, North of Scotland, North Tees, North Yorkshire, Nottingham, Oxford, Portsmouth, Rotherham, Sheffield, Shropshire, Somerset, South Birmingham, South East Scotland, South East Staffordshire, South Derbyshire, South Essex, South Lancashire, South West Scotland, Surrey, Warrington Halton St Helens & Knowsley, Warwickshire Solihull & Coventry, West Berkshire, West Devon, West London, West Suffolk, West Sussex, Wiltshire, Winchester, Wirral and Wycombe.

Million Women Study Steering Committee. Emily Banks, Valerie Beral, Ruth English, Jane Green, Julietta Patnick, Richard Peto, Gillian Reeves, Martin Vessey and Matthew Wallis.

Million Women Study Coordinating Centre. Simon Abbott, Naomi Allen, Miranda Armstrong, Angela Balkwill, Emily Banks, Vicky Benson, Valerie Beral, Judith Black, Anna Brown, Diana Bull, Benjamin Cairns, Kathy Callaghan, Karen Canfell, Dexter Canoy, James Chivenga, Barbara Crossley, Francesca Crowe, Dave Ewart, Sarah Ewart, Lee Fletcher, Toral Gathani, Laura Gerrard, Adrian Goodill, Jane Green, Lynden Guiver, Isobel Lingard,

Elizabeth Hilton, Sau Wan Kan, Carol Keene, Oksana Kirichek, Mary Kroll, Nicky Langston, Bette Liu, Maria-Jose Luque, Lynn Pank, Kirstin Pirie, Gillian Reeves, Andrew Roddam, Keith Shaw, Emma Sherman, Evie Sherry-Starmer, Helena Strange, Siân Sweetland, Alison Timadjer, Sarah Tipper, Ruth Travis, Xiaosi Wang, Joanna Watson, Lucy Wright, Owen Yang, Heather Young.
